# Case Report: Application of hepatitis B virus (HBV) deep sequencing to distinguish between acute and chronic infection

**DOI:** 10.12688/wellcomeopenres.16157.1

**Published:** 2020-10-14

**Authors:** Louise O. Downs, Anna L. McNaughton, Mariateresa de Cesare, M. Azim Ansari, Jacqueline Martin, Charles Woodrow, Rory Bowden, Jane Collier, Eleanor Barnes, Philippa C. Matthews

**Affiliations:** 1 Department of Infectious Diseases and Microbiology, Oxford University Hospitals NHS Foundation Trust, Oxford, OX3 9DU, UK; 2 Nuffield Department of Medicine, University of Oxford, Medawar Building, South Parks Rd, Oxford, OX1 3SY, UK; 3 Wellcome Centre for Human Genetics, Wellcome Centre for Human Genetics, Oxford, OX3 9DU, UK; 4 Department of Hepatology, Oxford University Hospitals NHS Foundation Trust, Oxford, OX3 9DU, UK; 5 Oxford NIHR BRC, Oxford University Hospitals NHS Foundation Trust, Oxford, OX3 9DU, UK

**Keywords:** Hepatitis B virus, reactivation, whole genome sequencing, prognosis, case report, IgM, IgG, acute hepatitis B.

## Abstract

Deep sequencing of the full-length hepatitis B virus (HBV) genome provides the opportunity to determine the extent to which viral diversity, genotype, polymorphisms, insertions and deletions may influence presentation and outcomes of disease. Increasing experience with analysis of HBV genomic data opens up the potential for using these data to inform insights into pathophysiology of infection and to underpin decision making in clinical practice. We here set out to undertake whole genome HBV sequencing from an adult who presented acutely unwell with a new diagnosis of HBV infection, and tested positive for both HBV anti-core IgM and IgG, possibly representing either acute hepatitis B infection (AHB) or chronic hepatitis B with an acute reactivation (CHB-AR). The distinction between these two scenarios may be important in predicting prognosis and underpinning treatment decisions, but can be challenging based on routine laboratory tests. Through application of deep whole-genome sequencing we typed the isolate as genotype-D1, and identified several minority variants including G1764A and G1986A substitutions in the pre-core promoter and pre-core regions, which support CHB-AR rather than AHB. In the longer term, enhanced deep sequencing data for HBV may provide improved evidence to distinguish between acute and chronic infection, to predict outcomes and to stratify treatment.

## Abbreviations

- AHB – Acute hepatitis B
- CHB – Chronic hepatitis B
- CHB-AR – Acute reactivation of chronic hepatitis B
- Anti-HBc-IgM – Hepatitis B anti-core IgM antibody
- HBsAg – Hepatitis B surface antigen
- TDF - Tenofovir disoproxil fumarate
- S/CO – Sample to cut-off ratio
- PEI units – Paul Ehrlich Institut units
- HBeAg – Hepatitis B e antigen
- NGS – Next generation sequencing

## Introduction

The course of hepatitis B virus (HBV) infection depends on the interplay between the virus and host immune system, with variable outcomes that include clearance, control, chronicity, cirrhosis and cancer. One other important disease manifestation is that of acute reactivation of chronic hepatitis B infection (CHB-AR), in which a previously quiescent virus causes a flare of hepatitis, typically characterised by a sudden rise in both serum alanine aminotransferase (ALT) and HBV DNA viral load ^1^. There are two different scenarios that may be referred to as CHB-AR: (i) patients with a consistently positive HBV surface antigen (HBsAg) test, but a baseline low or undetectable viraemia, followed by a sudden rise in HBV viral load (VL); (ii) patients in whom HBsAg has been cleared completely, but subsequently becomes detectable again in serum, usually in the context of immunosuppression ^2, 3^, associated with concurrent bacterial or HIV infection, in times of emotional or physical stress, and associated with pregnancy ^4– 6^.

Traditionally the presence of HBV anti-core IgM (anti-HBc-IgM) is considered a marker of acute hepatitis B (AHB) infection. However, with improvements in sensitivity of the IgM ELISA assay, low titres of IgM can now be detected in up to 70% of cases of CHB-AR, making it difficult to distinguish between the two syndromes ^7– 9^. Production of anti-HBc-IgM during CHB-AR may be due to alteration of antigenic epitopes leading to new antibody production, or increased display of core antigen due to hepatocellular lysis during CHB-AR ^10, 11^. One study estimated 27% of presumed AHB cases were in fact CHB-AR ^12^, and in endemic settings this may be even higher, with up to 70% of acute presentations being associated with chronic infection ^7^. The annual rate of CHB-AR has been estimated at 3.3% ^13^.

The distinction between AHB and CHB-AR can be prognostically important and can influence treatment approaches with antiviral agents. CHB-AR typically runs a less predictable course: in most cases, liver function tests (LFTs) and HBV DNA levels return to baseline but future flares of hepatitis can occur. CHB-AR may also be associated with severe hepatitis, occasionally leading to acute-on-chronic liver failure and death ^14^. After the acute flare has passed, the longer-term risks of cirrhosis and hepatocellular carcinoma associated with CHB are still present. In severe cases of CHB-AR, tenofovir disoproxil fumarate (TDF) is recommended, with some evidence that it reduces mortality ^15^. In contrast, AHB flares may resolve spontaneously and can result in HBsAg clearance without any specific treatment.

To distinguish AHB from CHB-AR, several studies have investigated quantitative anti-HBc-IgM ^7, 16, 17^. However, at present there is a lack of standardisation of commercial assays and no consensus as to a valid clinical threshold ^10, 18^. HBV DNA levels are typically higher in CHB-AR, but may be low or undetectable by the time new acute infection presents with clinical symptoms ^7, 10, 16^, due to the rapid immunological clearance of HBV DNA in AHB ^19^. Some studies suggest that higher ALT, aspartate aminotransferase (AST) and bilirubin (BR) levels point to AHB ^16, 20^, but this is not consistent ^7^. The combination of anti-HBc-IgM levels and quantitation of HBV DNA and/or HBsAg may become more sensitive and specific in distinguishing between AHB vs CHB-AR ^16, 21^, but this approach is not yet standardised.

As next generation sequencing (NGS) platforms become more accessible and affordable, there is potential to analyse HBV sequences to greater depth and accuracy to aid distinction of clinical syndromes and to inform treatment decisions ^22^. Multiple studies have focussed on HBV sequence polymorphisms associated with AHB versus CHB-AR, but most have focused on short regions of the genome using Sanger sequencing ^23– 26^, and there is limited insight into specific polymorphisms or features that might be helpful in discriminating between these two syndromes.

We here report the case of a patient with no known history of viral hepatitis presenting to hospital with an acute episode of hepatitis, and testing positive for HBsAg. Based on routine serological markers undertaken in the clinic, it was not possible to distinguish between AHB versus CHB-AR. We applied full length sequencing of HBV using Illumina deep sequencing to help identify any sequence polymorphisms that could help to distinguish between acute vs. chronic infection. The case illustrates the diagnostic difficulties that can be associated with the presentation of an acute flare of HBV and highlights the future potential for deep sequencing approaches to contribute to diagnosis, prognosis, and treatment decisions.

## Case report

A middle-aged man of Pakistani origin presented to a UK hospital with jaundice, dark urine, headache, fatigue and flu-like symptoms (Oxford viral hepatitis cohort, study ID: 1745). He had been unwell in the week preceding admission but had not sought prior medical attention. He was born in Pakistan but has been resident in the UK since childhood. He reported a history of HBV infection in an aunt in Pakistan; his wife and children in the UK all tested HBsAg-negative. He has no occupational risk factors for infection, does not drink alcohol, takes no medications, and is usually fit and active. He had recently returned from visiting Malaysia, but denied any risk factors for recent acquisition of HBV infection. On examination he was haemodynamically stable. He had conjunctival icterus, but there was no hepatosplenomegaly and his abdomen was soft and non-tender.

On further discussion, he reported several similar episodes of illness over preceding years, albeit less severe and without objective jaundice; on these grounds he had never previously presented for clinical review. Together with the absence of any risk factors for acute infection, this history raised the possibility of recurrent flares of hepatitis, leading us to consider whether this was a presentation of CHB-AR rather than AHB. We could not identify any precipitating factors leading to reactivation; specifically, there were no risk factors for immunocompromise.

At presentation, ALT was 608 IU/L (reference range 10–45 IU/L), BR 138 μmol/L (reference range 0–21 μmol/L), platelets 121 × 10 ^9^/L (reference range 150–400 × 10 ^9^/L) and prothrombin time 17.7 seconds (reference range 9–12 seconds). He tested positive for HBsAg, anti-HBc-IgM and IgG. Anti-HBc-IgM levels were 7.43 S/CO (reactive >1 S/CO corresponding to 50 PEI units). Hepatitis B e-antigen (HBeAg) was negative, hepatitis B e-antibody (anti-HBe) positive. HBV DNA was 5.5 log _10_ IU/mL. Hepatitis A, C, D, E and HIV were negative. Elastography score was elevated at 38.7kPa (normal range 2–7kPa). A liver ultrasound was normal with no intrahepatic or biliary duct dilatation and no evidence of liver fibrosis. At the time of presentation, the overall picture was deemed most consistent with acute HBV infection, with the elevated elastography score reflecting acute liver inflammation.

**Figure 1.**
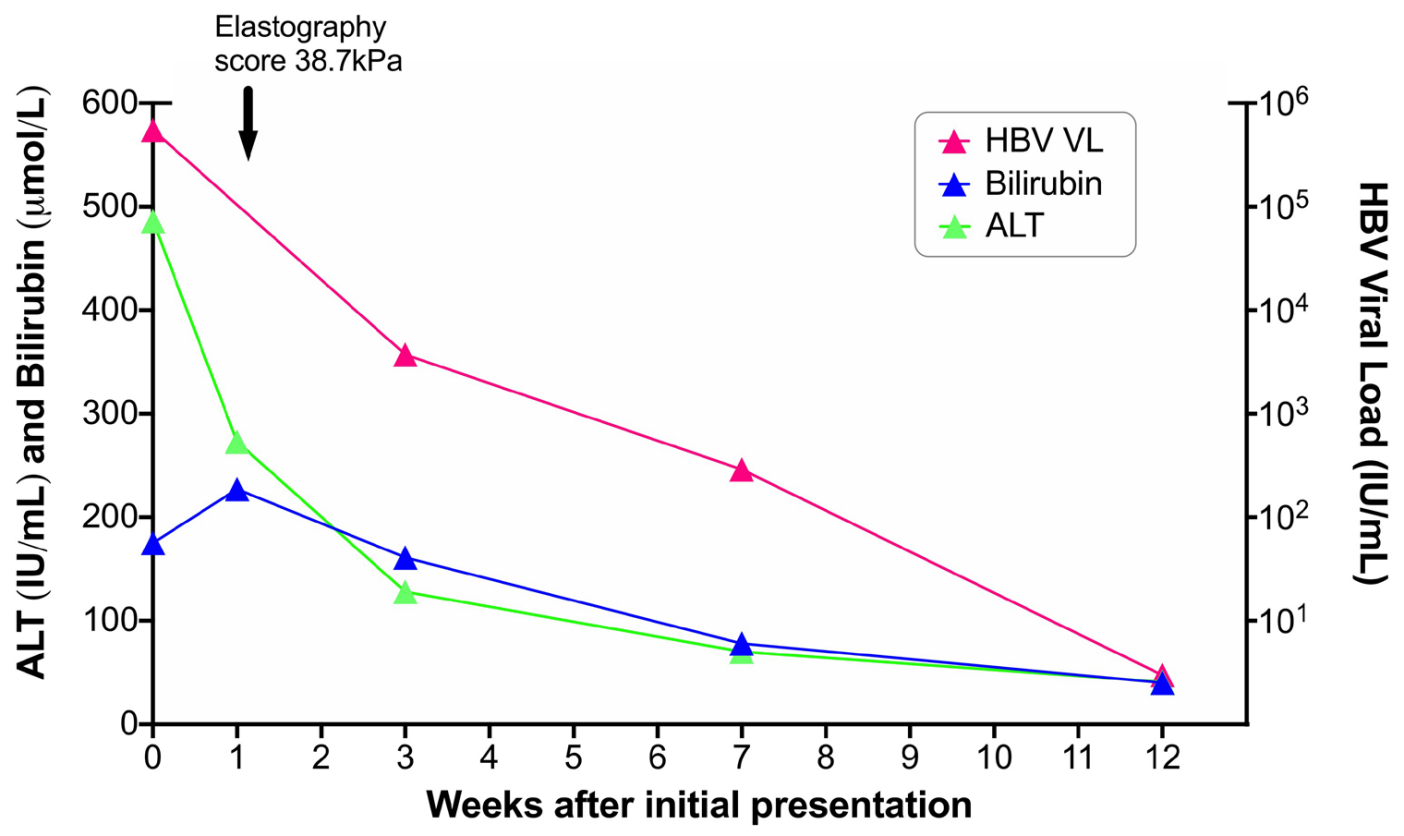
Laboratory timeline for an adult presenting with acutely deranged liver function tests in the setting of HBV infection. Anti-HBc IgM and IgG were both present throughout the timeline. Blood for whole genome sequencing was taken at presentation (week 0). Elastography score improved to 25.3kPa 6 months later then down to 20.7kPa at one year after initial presentation.

He received supportive care including intravenous fluids and close monitoring, and made a gradual clinical recovery over 2–3 months. Nucleos(t)ide analogue therapy was deferred on the grounds of gradual improvement and a chance of spontaneous clearance of HBsAg. At three months post-presentation, blood markers had all improved (HBV DNA 2.9 log _10_ IU/mL, ALT 41 IU/L, BR 40μmol/L ( Figure 1)). Anti-HBc-IgM was still reactive but had decreased to 3.37 S/CO. At six months post-presentation, HBsAg remained positive and he therefore now meets the case definition for chronic infection. He chose to remain off therapy, but is under close follow-up to allow us to continue to review the indications for antiviral treatment based on clinical progress and guidelines ^27^.

## Application of Illumina Sequencing of HBV

**Figure 2.**
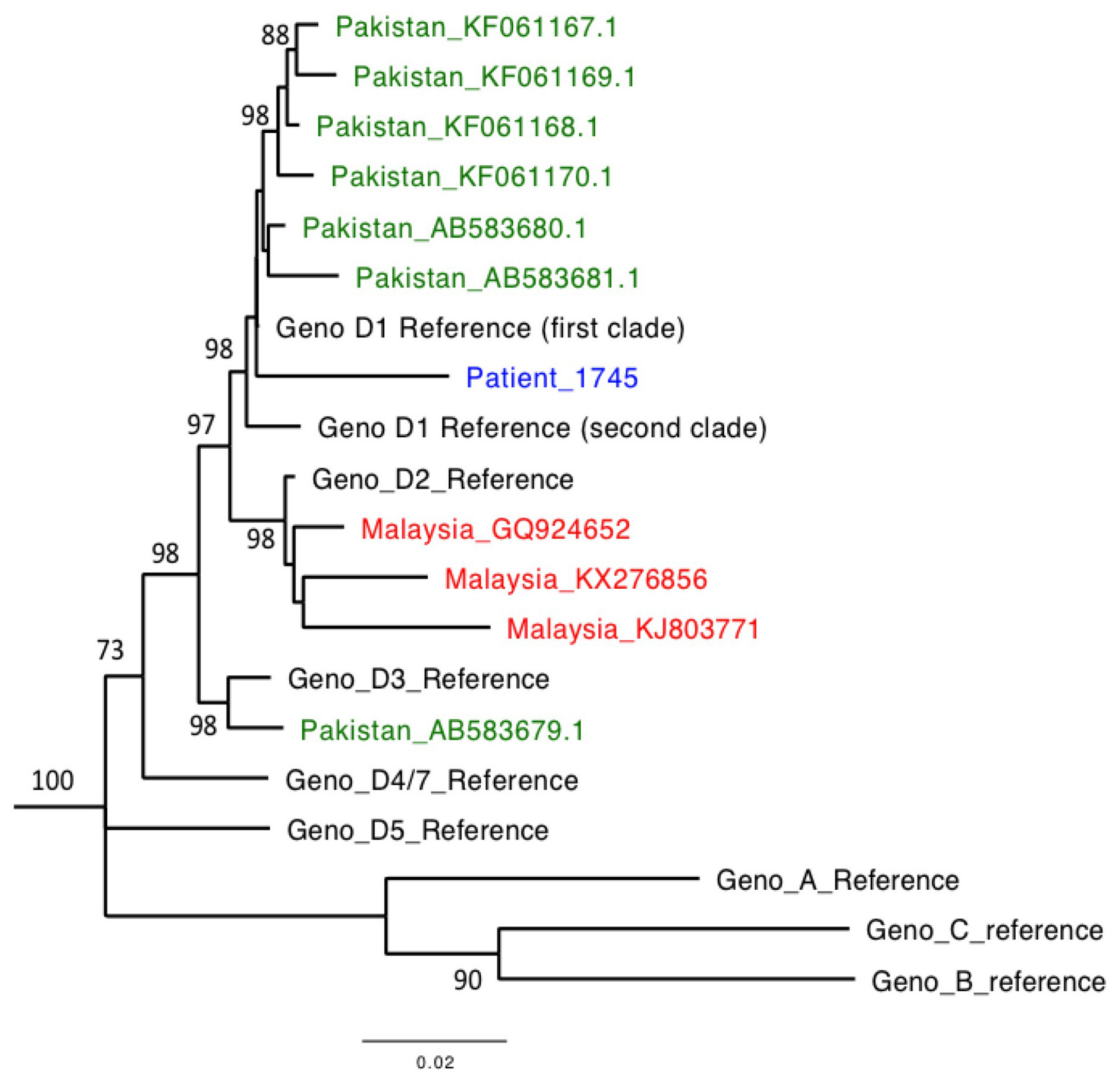
Maximum likelihood phylogenetic tree of HBV sequence derived from an adult male presenting acutely with HBV infection. Consensus HBV sequence for patient 1745 is shown (blue) alongside representative genotype and subtype reference sequences, and all full-length HBV sequences from Pakistan (green) and Malaysia (red) from GenBank. Reference sequences ^59^: A (X02763), B (GQ205440), C (KP017269), D1 (clade 1): KP322600, D1 (clade 2): JN040807, D2: KR905424, D3: KP322602, D5: KP322603, D4/7: FJ692533 (D4 and D7 cluster closely and this sequence is representative of the clade including both genotypes). Bootstrap replicates of 1000 generated using MEGA7. Bootstrap values of >70 are shown. Scale bar shows substitutions per site. The sequences in GenBank from Pakistan mapped to either subtype D1 (6/7 sequences) or D3 (1/7 sequences) whilst Malaysian sequences map to subtype D2 (3/3 sequences).

**Figure 3.**
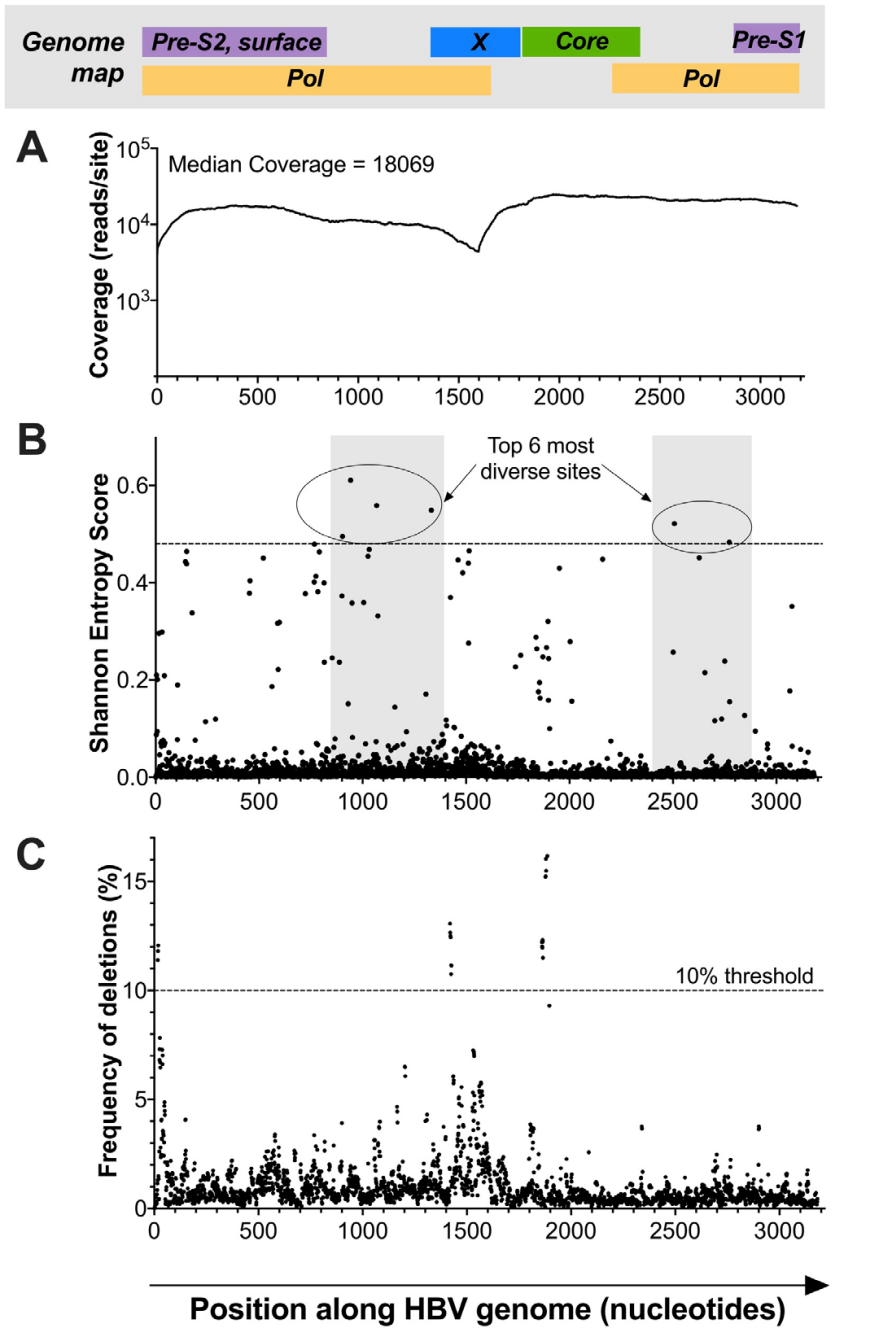
HBV genome map showing sequencing coverage, diversity and deletions for a genotype D sequence derived from a patient with acute biochemical hepatitis. In each case, the nucleotide position is shown on the x-axis, with the approximate positions of corresponding genes indicated in the bar at the top (adapted from McNaughton *et al.* ^22^). **A**: Illumina data showing read coverage (log scale) at each nucleotide site along the HBV genome. The drop in coverage around nt 1000-1600 corresponds to the single stranded portion of the genome. **B**: Shannon entropy calculated for each site along the genome. Shaded areas represent non-overlapping sections of the genome and the horizontal dotted line isolates the top six most diverse sites. **C**: Locations of minority variant deletions, with a threshold indicated at ≥10% to identify the deletions most likely to be relevant. Deletions shown at the start of the genome are likely to represent an artefact of the mapping (short reads derived from a circular genome have been mapped onto a linear construct).

Through application of Illumina sequencing to a baseline plasma sample (collected at the time of first presentation to hospital), we generated 1.28 million total reads, of which 74% mapped to HBV. The consensus sequence was 3182 nucleotides long and mapped to genotype D, clustering within subtype D1, making it most closely related to other published sequences from Pakistan ( Figure 2). After de-duplication, the median coverage was 18,069 reads per site ( Figure 3A). In this deep sequence dataset, 2.5% (80/3182) of nucleotide positions had a Shannon entropy score of >0.1 ( Figure 3B). These sites were evenly distributed across the genome with no obvious concentration in any particular gene.

In this deep sequence dataset, 2.5% (80/3182) of nucleotide positions had a Shannon entropy score of >0.1 ( Figure 2B). These sites were evenly distributed across the genome with no obvious concentration in any particular gene. Nucleotide diversity has been reported to be lower in acute HBV infection, possibly due to a small number of variants initiating new infection ^28, 29^. However, other reports have shown low viral diversity in CHB, mostly in the context of HBeAg positivity and high viral loads where immune pressure may be minimal ^30, 31^. The top six most diverse sites in this HBV genome were all located in non-overlapping regions in the polymerase gene, where mutations are least likely to have an impact on viral fitness and high diversity, as previously described ^22, 32, 33^.

The most prevalent minority variant mutations in our HBV sequences were G1896A, G1899A and G1764A (precore/core and basal core promotor sequences respectively) ( Table 1). G1896A converts codon 28 from tryptophan (TGG) to a stop codon (TAG) and terminates the translation of the HBeAg precursor ^35^. Mutations at amino acid level were also seen in surface antigen (V190A and T127P), both of which have been associated with CHB-AR in the context of immunosuppression ( Table 1). Several immune escape mutations have been identified in the literature in patients presenting with AHB, none of which were present in our sequences ^36^ ( Table 1). Minority variant deletions occurring in ≥10% of viral sequencing reads were detected at nt 1419–1426 and nt 1860–1865 in the RT/X-gene and pre-core genes respectively ( Figure 3C). Mutations at nt 1862 have previously been shown to impair genome replication ^37^, but deletions in either region have not been reported in the literature to date, to the best of our knowledge.

**Table 1.** Comparison of Hepatitis B virus (HBV) mutations described in the literature with those found in the HBV sequence of a patient presenting with acute biochemical hepatitis (patient 1745). Mutations are relating to chronic HBV with acute reactivation (CHB-AR), acute hepatitis B (AHB) and HBV associated acute on chronic liver failure. Mutations present in >10% of sequences are marked in bold, mutations present in <10% of sequences are not represented.

Gene (Protein)	Site	Polymorphisms reported in the literature	Disease Association and References	Sequencing Methods Used	Genes Sequenced	Genotype studied	Patient 1745 sequence
Precore/ core	1896	G1986A	CHB-AR during cytotoxic chemotherapy or SCT ^**^^23, 40^	Sanger ^23^ Sanger ^40^	Basal core promotor + precore ^23^ Surface, RT ^***^, precore ^40^	Not stated C/D	G1986: 66% **G1986A: 25%**
			Hepatitis B Related Acute-on-Chronic Liver Failure ^41^	Sanger	Basal core promotor, precore	B/C	
			Distinguish CHB-AR from AHB ^24, 42^	Enzyme linked assays ^24^ Sanger ^42^	Specific mutations only: G1896A + A1762T, G1764A ^24^ Pre-core ^42^	B/C Not stated	
			Comparing CHB with CHB-AR ^43^	Sanger	Basal core promotor, precore	B/C	
			CHB-AR and Fulminant hepatic failure during chemotherapy ^26^	Sanger	Full length genome	A/B/C	
	1899	G1899A	Hepatitis B-Related Acute-on-Chronic Liver Failure ^41^	Sanger	Basal core promotor, precore	B/C	G1899: 85% **G1899A: 14%**
			CHB-AR during chemotherapy or SCT ^40^	Sanger	Surface, RT, precore	C/D	
Precore Promoter Regions	1742	G1742A	CHB-AR during chemotherapy ^44^	Sanger	Basal core promotor, precore	Unknown	G1742: 95%
	1752	A1752G	CHB-AR during chemotherapy ^44^	Sanger	Basal core promotor, precore	Unknown	A1752: 98%
	1753	T1753V (C/A/G)	Hepatitis B-Related Acute-on-Chronic Liver Failure ^41^	Sanger	Basal core promotor, precore	B/C	T1753: 99%
			CHB-AR during chemotherapy ^44^	Sanger	Basal core promotor precore	Unknown	
	1754	T1754G	CHB-AR during chemotherapy ^44^	Sanger	Basal core promotor, precore	Unknown	T1754: 99%
	1762	A1762T	Hepatitis B-Related Acute-on-Chronic Liver Failure ^41^	Sanger	Basal core promotor, precore	B/C	A1762: 99%
			Distinguish CHB-AR from AHB ^24^	Enzyme linked assays	Specific mutations only: G1896A + A1762T, G1764A	B/C	
			CHB-AR ^25^	Sanger	Basal core promotor, precore	B/C	
			CHB-AR during chemotherapy ^44^	Sanger	Basal core promotor, precore	Unknown	
	1764	G1764A	Hepatitis B-Related Acute-on-Chronic Liver Failure ^41^	Sanger	Basal core promotor, precore	B/C	G1764: 84% **G1764A: 14%**
			Distinguish CHB-AR from AHB ^24^	Enzyme linked assays	Specific mutations only: G1896A + A1762T, G1764A	B/C	
			CHB-AR ^25^	Sanger	Basal core promotor, precore	B/C	
			CHB-AR during chemotherapy ^44^	Sanger	Basal core promotor, precore	Unknown	
	1766 1768	C1766T/T1768A double mutation & T1768A alone	Distinguish CHB-AR from AHB ^28^	Sanger	Surface, basal core promotor, precore, X-gene.	A/C/D	C1766: 97% T1768: 98%
	1799	G1799A/C1799G	CHB-AR during chemotherapy ^44^	Sanger	Basal core promotor, precore	Unknown	C1799: 98%
Surface Antigen (amino acid locations)	190	V190A	CHB-AR on immunosuppression ^45^	Sanger	Surface	D	**V190A: 97%**
	127	T127P	CHB-AR in aHSCT ^46^	Next generation deep sequencing	Full length genome	A/D/E	**T127P: 98%**
	118 120 128 133 145 172	T118A P120S/T A128V M133I G145R W172*	Immune escape mutations associated with AHB ^36^	Ultradeep pyro-sequencing	Surface and RT	A/D	**No mutations** **present**

**Stem cell transplant. *** Reverse transcriptase

There are clinical and laboratory features of this case suggestive of both AHB and CHB-AR, summarised in Table 2. Overall, we conclude that this patient is most likely to have presented with CHB-AR, on a background of infection early in life in Pakistan. 

**Table 2.** Summary of factors in the case of an adult presenting with an acute biochemical hepatitis favouring either AHB or CHB-AR.

	Evidence in support of AHB	Evidence in support of CHB-AR
Patient history	• Recent travel to Malaysia as a possible risk factor for HBV acquisition (prevalence estimated to be up to 9% ^47^). • No household members are HBsAg positive, and no (known) history of HBV infection in parents or siblings. • No precipitating factors identified for CHB-AR	• Patient’s own description of previous similar events, possibly representing previous episodes of CHB-AR. • Origin in Pakistan where HBV prevalence is estimated to be up to 4% ^48, 49^, with a history of infection in extended family members.
Routine clinical laboratory data	• Relatively high peak bilirubin level ^20^ (227 mol/L at highest).	• Some studies indicate an anti-HBc-IgM S/CO >10 is indicative of AHB ^17^. In this case, the S/CO level of 7.43 does not meet this threshold, suggesting CHB-AR may be more likely. • High HBV DNA level at diagnosis (5.5 log10 IU/ml). Studies have indicated this level of HBV DNA fits more with CHB-AR ^7, 16, 50^. • Relatively low rise in ALT (486 IU/ml at peak). • Patient remains HBsAg-positive six months after initial presentation. Since >90% of adults clear AHB infection, remaining HBsAg-positive is more in keeping with pre-existing CHB.
Deep sequencing data	• No polymorphisms associated with CHB-AR identified at consensus level	• Viral sub-genotype is D1, known to circulate in Pakistan ^51^, suggesting infection early in life, and supported by 6/7 of the other sequences from Pakistan being D1. • Presence of several minority variant mutations associated with CHB-AR and lack of mutations described to be associated with AHB.
	• Relative lack of nucleotide diversity in the HBV genome could be a feature of AHB due to a small number of HBV variants establishing new infection. Could also represent high viral load CHB where there is reduced immune selection and unregulated replication of conserved viral populations.	
	• The pattern of deletions seen here has not been reported as typical of association with either AHB or CHB-AR.	

AHB – acute hepatitis b virus infection; CHB-AR – acute reactivation of chronic hepatitis b virus infection; S/CO – sample to cut off ratio.

## Discussion

This case demonstrates how difficult it can be to distinguish between AHB and CHB-AR, and highlights inconsistencies in the way the term ‘reactivation’ is used. We have illustrated the potential application of whole genome deep sequencing data to identify sequence changes in the HBV genome that may be associated with a specific disease presentation.

Quantitative anti-HBc-IgM, HBsAg and HBV DNA are currently the best tools with which to distinguish between AHB and CHB-AR. However, further efforts are required to define diagnostic thresholds, and clinicians should consider other factors that might help to discriminate between AHB and CHB-AR. In the longer term, generation and publication of more HBV sequencing data are essential in order to improve insights into sequence motifs that are associated with specific clinical syndromes and outcomes of HBV infection. In order to meet the ambitious United Nations Sustainable Development Goals target for elimination of viral hepatitis as a public health threat by the year 2030 ^38^, action is required to improve the provision of treatment to those at highest risk of long-term complications and to reduce transmission at a population level. High resolution sequencing data is one way to advance our understanding of the outcomes and biology of infection, and to improve treatment stratification.

At present, it is difficult to come to any definite conclusion by analysing HBV sequence data from one patient at a single timepoint. Longitudinal sequencing would be helpful to detect any changes in HBV sequence, but the current sensitivity of NGS platforms limits our ability to generate sequences as HBV VL falls <10 ^4^ IU/ml ^30, 39^. Our patient had been unwell prior to hospital presentation, but we do not have clinical or biochemical data for this period, which could have shed further light on his illness (for example, changes in anti-HBc-IgM titre).

The sequence data available for comparison are extremely limited and there is a lack of representation from diverse geographical regions and different genotypes. Strikingly, in GenBank there are currently only seven full length HBV genome sequences from Pakistan (all genotype D), limiting the contribution made by phylogenetic analysis to help discern the origin of our patient’s HBV infection.

## Conclusion

Current laboratory methods deployed in routine clinical practice may not reliably distinguish between CHB-AR versus AHB. However, this distinction may be important in prognostication and planning appropriate clinical care. To date, HBV sequence data relating to the distinction of AHB and CHB-AR mostly consists of individual genes at the Sanger sequencing level, particularly core and pre-core genes ( Table 1), and we have shown how this approach can help to inform a better understanding of a clinical case. As more deep sequence data are generated with better representation of diverse genotypes, viral sequence motifs may emerge that allow further improvements to be made in determining this distinction, with the potential to improve insights into prognosis and underpin decisions about antiviral therapy. 

## Methods

Plasma samples were taken at the time of index presentation to hospital. We extracted total nucleic acid from 0.5ml plasma, using the NucliSENS automated magnetic nucleic acid extraction system (cat. No. 280140, BioMérieux), eluting into 25µl elution buffer, as per the manufacturer’s instructions. We undertook a completion-ligation reaction, incubating the partially double stranded (ds)DNA genome with a T4 ligase and T4 polymerase (cat. No. M0202S and M0203S respectively, both supplied from New England Biolabs) at 30ºC for 45 minutes, in order to generate fully dsDNA HBV genomes, as previously described ^30, 52^. Nucleic acid was then purified using Agencourt RNAClean XP magnetic beads (cat. No. A63987, Beckman Coulter). We generated sequencing libraries using the NEBNext Ultra II FS DNA Library Prep Kit for Illumina (cat. No. E7805L, New England Biolabs) according to manufacturer’s instructions and enriched for HBV DNA using a target-enrichment workflow modified from the SeqCap EZ (Roche) protocol, using custom-designed pangenotypic HBV probes spanning the full-length viral genome ordered from IDT (xGen Lockdown Probes). Probe sequences are not yet published but we welcome approaches for collaborations using this method; see further details in ‘data availability’ section.

We sequenced libraries on an Illumina MiSeq platform using a v3 300-bp paired end kit, then demultiplexed paired-end Illumina reads and removed poor-quality bases and adaptor sequences ( QUASR v7.01 and Cutadapt v1.7.1 software ^53, 54^). Human reads were removed by mapping to the human reference genome, hg19 using bowtie2 v2.2.4 ^55^. We used BWA mem ^56^ to map non-human reads to HBV genotype A-H consensus reference sequences, derived from 4500 whole genomes stored on HBVdb ^57^ (sequences available from https://github.com/hr283).

We generated a maximum likelihood phylogenetic tree using MEGA7 ^58^ with bootstrap replicates of 1000. Based on our patient’s travel history to Pakistan and Malaysia, we downloaded all full-length HBV genome sequences from Pakistan and Malaysia from GenBank (April 2019) ^59^. We included all those from Pakistan (n=7) and identified genotype D sequences from Malaysia (n=3) (see Figure 2).

To assess viral diversity, we aligned our sequence against the HBV sequence AX02763, (genotype A reference strain widely used for numbering), with the EcoR1 site as nucleotide 1. We calculated nucleotide variance using Shannon entropy for each site across the HBV genome to quantify viral diversity. We assessed for the presence of polymorphisms, insertions and deletions (indels) at consensus level and then examined the deep sequencing reads for evidence of minority variants. We set a threshold of ≥10% frequency to identify the most relevant polymorphisms, deletions and indels. We searched the literature to identify HBV sequence polymorphisms associated with AHB, CHB-AR and HBV-related acute-on-chronic liver failure (summarised in Table 1). We compared polymorphisms found in HBV sequences from patient 1745 with those we had identified from the published literature ( Table 1).

## Ethics

Approval for this work was provided by Oxford Research Ethics Committee A (reference 09/H0604/20). Written informed consent for publication of their clinical details was obtained from the patient.

## Consent

We obtained written consent from this patient both for enrolment into the Oxford Hepatitis Cohort Study, and for his specific agreement to publication of this individual case report.

## Data availability

### Underlying data

Sequence data has been submitted to GenBank: Accession number MT114169.

### Probe sequences

The sequences of the custom-designed HBV enrichment probe set used for HBV enrichment are not currently available due to potential collaboration with industry for commercial development. However, we welcome applications for collaboration using this method, which we will consider on a case-by-case basis, accounting for the nature of the research question, sample set (available material, number of samples to be sequenced), and allocation of resources required for the project (funding and manpower). Please contact philippa.matthews@ndm.ox.ac.uk or azim.ansari@ndm.ox.ac.uk for further information.

### Reporting guidelines

Figshare: CARE checklist for ‘Application of hepatitis B virus (HBV) deep sequencing to distinguish between acute and chronic infection’ https://doi.org/10.6084/m9.figshare.12649196.v1 ^60^

Data are available under the terms of the Creative Commons Attribution 4.0 International license (CC-BY 4.0).
