# Supplementary material for: Case Report: Application of hepatitis B virus (HBV) deep sequencing to distinguish between acute and chronic infection
Source: Wellcome Open Res. 2021 Jan 25;5:240. Originally published 2020 Oct 14. [Version 2] doi: 10.12688/wellcomeopenres.16157.2 (PMC7802106; doi:10.12688/wellcomeopenres.16157.2)
Supplement: Peer review history

# Peer review: Reviewer response for version 1

**DOI:** 10.21956/wellcomeopenres.17738.r42009

**Published:** 2021-01-11

**Authors:** Simon B Larsson, Johan Ringlander

**Affiliations:** 1 Department of Infectious Diseases, Institute of Biomedicine, University of Gothenburg, Gothenburg, Sweden

Downs *et al.* propose in this article a method to distinguish between acute hepatitis B infection and chronic hepatitis B infection with acute reactivation. They use whole genome sequencing to identify sequence polymorphisms representative for either scenario. A well-written and relevant article.

 To separate between recently acquired hepatitis B causing acute hepatitis with a beneficial prognosis and acute-on-chronic hepatitis, with a more complicated and usually worse prognosis, is sometimes difficult. The potential use of deep sequencing has to our knowledge not been presented like this before.

 Similar methods using a probe-based enrichment of HBV and downstream sequencing with Illumina has been described before and used in many different HBV projects. This seems to be a sensitive and robust method. However, if using PCR for enrichment, the sensitivity might be increased. The potential benefit of the probe-based method might be a lower risk of PCR induced errors.

 The conclusion that discrimination between acute and acute-on-chronic hepatitis B can be made based on pre-core mutations is interesting. In this case, many anamnestic details speak for an acute-on-chronic form, but there are cases where it is more unclear. It is interesting that this patient has minority populations containing pre-core mutations associated with HBeAg loss, which might indicate that they have arisen within this host. However, it is unknown to what degree subpopulations are inherited from the transmitting host and if/how they might prevail in the new host after transmission. From clinical experience, we know of one case with acute hepatitis B, the patient had previous serological records supporting HBV naivety, that had a majority virus population containing G1764A and G1896A pre-core mutations from start, most probably inherited from the transmitting host. However, if such subpopulations are rare or non-existing in newly infected patients presenting with acute hepatitis B, this method may still prove to be useful.

 Minor comments:

 It is not stated whether the other family members were positive for anti-HBs and/or anti-HBc.

 Formal remarks:

 In abstract and table 1. Position “1986” is stated when the position should be “1896”.

Are enough details provided of any physical examination and diagnostic tests, treatment given and outcomes?

Yes

Is the case presented with sufficient detail to be useful for other practitioners?

Yes

Is sufficient discussion included of the importance of the findings and their relevance to future understanding of disease processes, diagnosis or treatment?

Yes

Is the background of the case’s history and progression described in sufficient detail?

Yes

Reviewer Expertise:

Hepatitis B virus replication and integration (including real-time PCR, sequencing, NGS with focus on liver biopsies and explants form patients with HBV).

We confirm that we have read this submission and believe that we have an appropriate level of expertise to confirm that it is of an acceptable scientific standard.

# Peer review: Reviewer response for version 1

**DOI:** 10.21956/wellcomeopenres.17738.r40913

**Published:** 2020-11-12

**Authors:** Dennis Armando Bertolini

**Affiliations:** 1 Department of Clinical Analysis and Biomedicine, State University of Maringa, Maringa, Brazil

Hepatitis B really deserves special attention, especially in patients who develop the chronic form and those who have recovered from the infection, since they may have the hidden form of the virus. The criteria for differentiation from acute hepatitis B and acute reactivation from chronic hepatitis B lack definitions in order to have a better prognostic and treatment assessment. This case report intends to propose the use of the complete sequencing of the HBV genome as another criterion to assist in the definition of the situations presented above, analyzing the mutations and / or deletions that may be present in the genome that would make this definition more secure. The case in question presented the conditions to suspect an acute reactivation of chronic infection in view of the patient's history, symptoms presented, results of laboratory parameters and the use of specific therapy, which was well reported by the authors.

 The discussion formulated by the authors highlights the difficulty in defining acute hepatitis B and acute reactivation of chronic hepatitis B. The laboratory parameters HBsAg, anti-HBc IgM and DNA-HBV assist in this definition, but other information is needed to have greater security in discriminating the situations presented. They suggest a longitudinal study of patients with these characteristics, however the literature highlights the lack of sensitivity of the complete genome sequencing methodology when the viral load is below <104 IU / mL. The absence of clinical and biochemical information about the patient in question during the period when he was ill, made it difficult to reach a conclusion. The absence of complete HBV sequences in the literature to make a more detailed comparison was also a problem so that the result could be totally reliable. They conclude by suggesting that more studies be done with this methodology to obtain more information about the HBV genome in an acute infection and in an acute reactivation of chronic hepatitis B, including with a greater representativeness of the other HBV genotypes. The absence of complete HBV sequences in the literature to make a more detailed comparison was also a problem so that the result could be totally reliable. Therefore, I am in favor of indexing this case report.

Are enough details provided of any physical examination and diagnostic tests, treatment given and outcomes?

Yes

Is the case presented with sufficient detail to be useful for other practitioners?

Yes

Is sufficient discussion included of the importance of the findings and their relevance to future understanding of disease processes, diagnosis or treatment?

Yes

Is the background of the case’s history and progression described in sufficient detail?

Yes

Reviewer Expertise:

My area of ​​experience is human virology, mainly HIV, Hepatitis B and C, arbovirus and SARS-CoV-2. In addition, I work with epidemiology, genotyping, resistance to treatment and development of diagnostic methodologies

I confirm that I have read this submission and believe that I have an appropriate level of expertise to confirm that it is of an acceptable scientific standard.
